# A system for automated analysis of conductance correlations involved in recovery of electrical activity after neuromodulator deprivation in stomatogastric neuron models

**DOI:** 10.1186/1471-2202-15-S1-P41

**Published:** 2014-07-21

**Authors:** Atish Malik, Astrid A Prinz, Tomasz G Smolinski

**Affiliations:** 1 Department of Computer and Information Sciences, Delaware State University, Dover, DE 19901, USA; 2 Department of Biology, Emory University, Atlanta, GA 30322, USA

## 

Coregulation of ionic current levels, and specifically the changes that appear to take place in such relationships in response to deafferentation (*i.e.*, neuromodulator deprivation), is thought to be one of the main explanations behind the phenomenon of recovery of electrical activity exhibited by stomatogastric (STG) neurons subjected to deafferentation [3]. Here, we are proposing an automated software system that allows for analysis of conductance correlations involved in recovery of electrical activity after deafferentation in STG neuron models. The system utilizes multi-objective evolutionary algorithms (MOEA) to explore the parameter space of conductance-based neuronal models in search of those models that exhibit activity resembling that of neurons in presence of neuromodulation, despite being simulated without it [1]. The system considers such models to represent “recovered” neurons, and compares the correlations discovered in a population of those models to relationships exhibited by “control” model neurons (*i.e.*, those simulated with neuromodulators present). After the MOEA-based model construction is finished, the system automatically generates appropriate scatter plots, quantifies the relationships, finds differences between the two populations of models, and calculates the statistical significance of those differences.

As a case study, we apply our system to the analyses of models of two very important STG neurons: the anterior burster (AB) and pyloric dilator (PD), proposed in [2]. In addition to demonstrating the applicability of this approach, we discuss interesting insights into the phenomenon of function recovery that all seem to involve the delayed rectifier (Kd) current.

## References

[B1] Malik A Shim K Prinz A Smolinski TG Multi-objective evolutionary algorithms for analysis of conductance correlations involved in recovery of bursting after neuromodulator deprivation in lobster stomatogastric neuron models BMC Neurosci 2014 15 Suppl 1 P370

[B2] Soto-Treviño C Rabbah P Marder E Nadim F Computational model of electrically coupled, intrinsically distinct pacemaker neurons J Neurophysiol 2005 15 590 604 10.1152/jn.00013.2005 15728775 PMC1941697

[B3] Temporal S Desai M Khorkova O Varghese G Dai A Schulz DJ Golowasch J Neuromodulation independently determines correlated channel expression and conductance levels in motor neurons of the stomatogastric ganglion J Neurophysiol 2012 15 718 727 10.1152/jn.00622.2011 21994267 PMC3349629

